# Editorial

**Published:** 1870-04

**Authors:** 


### Editorial.

Illinois State Medical Society. — We call the attention of our readers to the Secretary's notice of the next annual meeting of the State Society, which is to be held in Dixon, on the *third* Tuesday in May next. We hope there will be a general representation of the profession throughout the State, and that every committee will be ready to report promptly.

---

Chicago Medical College Commencement. — The exercises of the public Commencement of the Chicago Medical College, Medical Department of the North-western University, were held on the 21st and 22d of March. We copy the following notice of the occasion from the *Daily Tribune:*—

The excellent charge of Professor Haven to the graduates will be given in full in our next issue.

The Eleventh Annual Commencement of the Chicago Medical College was held yesterday afternoon, at No. 1015 State Street. There was a large attendance of the relatives and friends of the graduates. The Chair was occupied by Rev. Erastus O. Haven, President of the North-western University. Prayer was offered by Rev. D. Lord.

Dr. N. S. Davis, President of the College, delivered a short address about the progress of the institution.

Dr. Edmund Andrews, Secretary of the College, presented certificates to undergraduates, and also to students who did hospital service.

The premium for the best thesis was awarded to H. F. Blackman, and for the second best to C. W. Earle.

The premium for the best examination on orthœpedic surgery was awarded to Lucius Dillie.

Rev. E. O. Haven then addressed the graduating class, stating, that by the authority vested in him as President of the North-western University, with which the Chicago Medical College was associated, and with the approval of the Faculty of the College, he conferred upon them their degrees.

The following named gentlemen were given diplomas:—

*Ordinary Degrees*—Francis Homer Blackman, John Wesley Boggiss, John Waldo Booth, Reuben Willis Bower, Henry Harrison Clark, Lester Curtis, Lucius Dillie, Charles Warrington Earle, Maurice Edwards, George Washington French, John Hall Hudson, Clark Israel Miller, George Franklin Nealley, George Washington Pattee, Willard Parker Pike, Stephen William Ranson, Cyrus Clay Reichard, Albert Louis Shay, William Moffat Stratton, Charles Eliot Wing.

*Ad Eundem Degrees* — Darwin L. Manchester, M.D., Mary H. Thompson, M.D., D. W. Young, M.D., D. Newcomb, M.D.

*Honorary Degrees* — J. M. Jenkins, B. L. Steel, T. F. Mayhem.

President Haven addressed the graduating class, dwelling upon the necessity of education to physicians, and the necessity of all educated men knowing something of medicine. Physicians should be liberal in their natures. There should be no learned ignorance. To encourage the study of medicine, the North-western University had given $15,000, to build a home for the Chicago College of Medicine. [Applause.] They would try to make the union reciprocal in its advantages. Comparing the mode of studying medicine in Europe and America, he prophesied that our system would in the end prove the best. They should get rid of quackery. [Applause.] Doctors should be the prosecutors of science and free thought. They had received diplomas from what he believed to be the best medical college in the United States. Let them go out and maintain the honor and dignity of their profession. They should never forget their *alma mater*. When they returned to the next commencement, their *alma mater* would be in a new building, so, if they had any valedictory tears to shed after the old building, they had better prepare to shed them now. They should be Christians; they should be men. He welcomed them to the ranks of medicine, and hoped they would always honor it. The learned gentleman was loudly applauded.

Dr. C. C. Reichard responded in behalf of the class. He thanked the Faculty for their kindness, and promised, on behalf of himself and comrades, never to forget their *alma mater*. [Applause.]

Professor H. A. Johnson, M.D., delivered the valedictory address. He bade them farewell as pupils, and welcomed them as doctors and associates. They should search always for the truth, as the truth would make them free. It would release them from the tyranny of prejudice. They should reject nothing because it was old, nor accept anything because it was new. With clean hands and pure hearts they should minister at the altar of life. The profession of medicine existed for society. Doctors were not necessarily a necessary nuisance any more than ministers, merchants, or any others. Their duties were to raise humanity from physical ills. They should care for men's bodies and minds, and be especially careful to enforce, as far as they could, the laws of hygiene, so that they might become conservators, as well as restorers, of the public health. They should always be ready to assist hospitals, and, to Christian hearts, the widow and the orphan never appealed in vain. They should be always on the alert to do their duties promptly. They should cultivate their hearts and its tenderest sympathies. Professional secrets should be held inviolable. An interesting address was concluded by an exortation to the graduates to study always to do their duty, and to promote the welfare of their fellow-men. The address was loudly applauded.

Then there was a song entitled "*Alma Mater O*," the joint production of Messrs. Lathrop and Earle. It was sung to the tune of the "Wearing of the Green," and created much enthusiasm.

The President pronounced the benediction, and the pleasant exercises were over.

### THE PRESIDENT'S RECEPTION.

In the evening, a reception was held at the hospitable mansion of Dr. N. S. Davis, No. 797 Wabash Avenue. The Professors of the North-western University, of the Medical College, the graduates, the students and their friends, were in goodly numbers. There was a pleasant time all round.

Illinois State Medical Society:

Illinois State Medical Society, Office of Permanent Secretary,
*181 West Madison Street, Chicago, Ill., March 22, 1870.*

The twentieth annual session will be held in Dixon, Lee County, on the third Tuesday in May, 1870, at 10 A.M.

The following committees are expected to report:

On the Practice of Medicine, Dr. C. Goodbrake, of Clinton, Chairman.

On Surgery, Dr. Moses Gunn, of Chicago, Chairman.

On Obstetrics, Dr. Elias Wenger, of Gilman, Chairman.

On Drugs and Medicines, Dr. Charles Hunt, of Dixon, Chairman.

On Necrology, Dr. J. H. Hollister, of Chicago, Chairman.

Special Committees:

On Criminal Abortions, Dr. DeLaskie Miller, of Chicago, Chairman.

On Ophthalmology, Dr. J. S. Hildreth, of Chicago, Chairman.

On Pulmonary Tuberculosis, Dr. J. P. Ross, of Chicago, Chairman.

On the Use of Plaster Paris in Fractures, Dr. R. G. Bogue, of Chicago, Chairman.

On Otology, Dr. Samuel J. Jones, of Chicago, Chairman.

On Arrangements, Drs. Everett, Philiips, and Hunt, of Dixon, Dr. J. C. Corbus, of Amboy, and Dr. E. P. Cook, of Mendota.

On Medical Education, Dr. N. S. Davis, of Chicago, Chairman.

Secretaries of all medical organizations are requested to send lists of their delegates to the Permanent Secretary, as soon as elected. Medical societies are entitled to one delegate for every five members. Any respectable physician, unable to attend as a delegate, or permanent member, may attend as a member by invitation, on the recommendation of the committee of arrangements. T. D. FITCH.

---

Kansas Medical Law.—We have been kindly furnished, by Dr. J. Parsons, with the following copy of a law just passed by the Legislature of the young State of Kansas:

"An Act to protect the people of Kansas from empiricism, and to elevate the standing of the medical profession.

"Be it enacted by the Legislature of the State of Kansas:

"Section 1. That it shall be unlawful for any person within the limits of the State of Kansas, who has not attended two full courses of instruction, and graduated in some respectable school of medicine, either of the United States, or of some foreign country, or who cannot produce a certificate of qualification from some State or county medical society, and is not a person of good moral character, to practice medicine in any of its departments, for reward or compensation, for any sick person within the State of Kansas; provided, that in all cases, when any person has been continuously engaged in the practice of medicine for a period of ten years, or more, he shall be considered to have complied with the provisions of this act, and that where persons have been in continuous practice of medicine for five years or more, shall be allowed two years in which to comply with such provisions.

"SEC. 2. Any person living in the State of Kansas, or any person coming into said State, who shall practice, or attempt to practice medicine, in any of its departments, or perform, or attempt to perform any surgical operation upon any person within the limits of said State, in violation of section one of this act, shall, upon conviction thereof, be fined in not less than fifty nor more than one hundred dollars for such offense, and upon conviction for a second violation of this act, shall, in addition to the above fine, be imprisoned in the county jail of the county in which said offense shall have been committed, for the term of thirty days, and in no case wherein this act shall have been violated, shall any person so violating, receive a compensation for services rendered; provided, that nothing herein contained shall in any way be construed to any person practicing dentistry.

"SEC. 3. This act shall take effect and be in force from and after its publication according to law."

---

ARSENIC IN PHTHISIS.—Mr. T. F. Sanger (*Lancet*) records his experience in a considerable number of cases, in favor of arsenic in consumption. He says, "In almost every case, the patient derived benefit from it, and several that I thought were hopeless, improved beyond my most sanguine expectations." A large majority of the patients were in the incipient stage; they were given five minims of Fowler's solution twice a day, soon after meals, with a drachm of iron wine, or five grains of the citrate of iron and quinine, or from fifteen to twenty grains of the hyposulphite of soda. It may be added that the locality where the cases were treated is a most favorable one for consumptives, having a chalk or porous sand soil, rendering it remarkably dry.—*Pacific Medical and Surgical Journal.*

### Mortality for the Month of February, 1870.

### CAUSES OF DEATH,

| | |
|---|---|
| Accident, burned by clothes tak'g fire | 1 |
| " by fall | 2 |
| " naphtha | 1 |
| " asphyxia, f'm gas | 1 |
| " " by screw in throat | 1 |
| " crushed, lumber | 1 |
| " injured by knife in plaining-mill | 1 |
| " poisoned by belladonna | 1 |
| " " or designedly | 3 |
| " railroad | 5 |
| Abscess of spine, near 6 and 7 vertebra | 1 |
| Anasarca | 1 |
| Aneurism, renal artery | 1 |
| Angina pectoris, | 1 |
| " | 1 |
| Apoplexy | 3 |
| Arachnitis | 2 |
| Asphyxia | 1 |
| Atelectasis pulmonum | 2 |
| Births, premature | 16 |
| " still | 37 |
| Bowels, ulceration | 1 |
| " intussusception | 1 |
| Brain, congestion of | 2 |
| " inflammation | 3 |
| " membrane, inflammation | 1 |
| " dropsy | 1 |
| " softening | 1 |
| Bronchitis | 10 |
| " capillary | 5 |
| Cholera infantum | 1 |
| Consumption | 38 |
| Convulsions | 46 |
| " puerperal | 1 |
| Croup | 20 |
| " diphtheretic | 1 |
| " membranous | 1 |
| Cyanosis | 1 |
| Debility | 5 |
| Development imperfe't | 2 |
| Diphtheria | 15 |
| Dropsy, abdominal | 2 |
| Dysentery | 1 |
| Encephalitis | 1 |
| Endocarditis | 1 |
| Enteritis | 5 |
| " and rheumatism, | 1 |
| Fever, intermittent, and inflammation of brain, | 1 |
| " puerperal, | 7 |
| " " and pyæmia | 1 |
| " remittent | 3 |
| " " convulsions | 1 |
| " scarlet | 28 |
| " " complications | 11 |
| " " malignant | 4 |
| " typhoid | 8 |
| Gastromalaxia | 1 |
| Glands, chronic enlargement, | 1 |
| Heart disease | 1 |
| " " and congestion of lungs | 1 |
| " " and emphyzema, lungs | 1 |
| " dropsy | 1 |
| " rheumatic inflam & œdema, lungs | 1 |
| " organic disease, | 1 |
| " valvular disease | 6 |
| " dropsy | 1 |
| Hip, disease | 2 |
| Hernia, inguinal | 1 |
| Hydrothorax | 2 |
| Hydrocephalus | 4 |
| " and carbuncle, | 1 |
| Inanition | 8 |
| Kidneys, Bright's disease of | 2 |
| " cancer | 1 |
| Laryngitis | 5 |
| Liver, cancer | 1 |
| " encephaloid disease | 1 |
| " inflammation | 1 |
| Lungs, congestion | 7 |
| " feited abscess | 1 |
| " hemorrhage | 1 |
| Measles | 4 |
| " & complications | 5 |
| Meningitis | 4 |
| " cerebro-spinal | 5 |
| " tubercular | 2 |
| Metritis, puerperal | 1 |
| Old age | 4 |
| Œdema pulmonum | 2 |
| Paralysis general and convulsions | 1 |
| Peritonitis | 1 |
| " puerperal | 1 |
| Phrenitis | 2 |
| Pleurisy, chronic | 1 |
| Pneumonia | 25 |
| " broncho | 2 |
| " & complications | 3 |
| " typhoid | 4 |
| Pyæmia | 2 |
| Small-pox | 1 |
| Spine, inflammation of | 1 |
| Stomach & Bowels ulceration & diarrhœa | 1 |
| " ulceration | 1 |
| " hemorrhage | 1 |
| Suicide, poison | 1 |
| " hanging | 1 |
| Tabes mesenterica, | 7 |
| Teething | 4 |
| " & complications | 3 |
| Tongue, cancer | 1 |
| Typhlitis | 1 |
| Uræmia | 1 |
| " from chronic tumor of prostate gland | 1 |
| Uterus, cancer of | 1 |
| Whooping-cough | 5 |
| " & complications | 6 |
| Total | 420 |

### AGES.

| | |
|---|---|
| Under 1 | 123 |
| 1 to 2 | 64 |
| 2 to 3 | 33 |
| 3 to 4 | 17 |
| 4 to 5 | 16 |
| 5 to 10 | 25 |
| 10 to 20 | 23 |
| 20 to 30 | 40 |
| 30 to 40 | 28 |
| 40 to 50 | 18 |
| 50 to 60 | 16 |
| 60 to 70 | 10 |
| 70 to 80 | 5 |
| 80 to 90 | 2 |
| Total, | 420 |

| | | |
|---|---|---|
| Males, 212 | Females, 208 | Total, 420 |
| Single, 330 | Married 90 | Total, 420 |
| White, 416 | Colored, 4 | Total, 420 |

COMPARISON.

| | | |
|---|---|---|
| Deaths in Feb. 1870, 420 | Deaths in Feb. 1869, 333 | Increase, 87 |
| Deaths in Jan'y, 1870, 483 | Decrease, 63 | |

NATIVITY.

| | | | | | |
|---|---|---|---|---|---|
| Bohemia | 2 | England | 9 | Scotland, | 3 |
| Canada | 4 | France | 2 | Sweden | 7 |
| Chicago, Native | 65 | Germany | 46 | Switzerland | 1 |
| Chicago, Foreign | 180 | Holland | 2 | | |
| U. S., other parts | 59 | Ireland | 32 | Total, | 420 |
| Denmark | 2 | Norway | 6 | | |

MORTALITY BY WARDS FOR THE MONTH.

| Wards. | Mortality. | | Mortality. |
|---|---|---|---|
| 1 | 6 | 16 | 27 |
| 2 | 12 | 17 | 32 |
| 3 | 16 | 18 | 34 |
| 4 | 9 | 19 | 13 |
| 5 | 14 | 20 | 14 |
| 6 | 29 | Accidents | 17 |
| 7 | 18 | County Hospital | 8 |
| 8 | 29 | Manslaughter | 1 |
| 9 | 37 | Mercy Hospital, | 1 |
| 10 | 8 | St. Luke's Hospital | 1 |
| 11 | 16 | St. Joseph Orphan Asylum, | 5 |
| 12 | 14 | Protestant Orphan Asylum | 1 |
| 13 | 6 | Suicides | 2 |
| 14 | 8 | | |
| 15 | 42 | Total, | 420 |

An Antidote to Phosphorus.—M. Personne, a well-known chemical experimenter of Paris, has just communicated to the Academy of Sciences some important facts in connection with poisoning by phosphorus. He believes that the fatal effects of phosphorus, on the human organism, are occasioned by the fact that the substance rapidly absorbs the oxygen of the blood whilst burning in that liquid; and in order to demonstrate this he performed a series of experiments with pyrogallic acid, which has quite a different composition from phosphorus, but possesses the like property of absorbing energetically the oxygen of air when placed in contact with an alkali. The results have shown that the administration of the acid to dogs, in doses varying from two to four grammes, produces the same effects, and is attended by the same lesions, as follow the use of phosphorus. As a practical consequence, and in order to prevent the baneful effects of phosphorus, or pyrogallic acid, M. Personne advises the use of turpentine, which must prevent the internal combustion, since, as is well known, it opposes the rapid absorption of atmospheric oxygen by phosphorus, even at a low temperature. This theoretical view has just been applied with complete success in two cases of poisoning by phosphorus under treatment in the Paris hospitals. The patients rallied completely through the timely administration of essence of turpentine. Cases of poisoning by phosphorus are so frequent that it is extremely important to become acquainted with an antidote which has hitherto furnished the best results.—*Medical & Surg. Reporter.*

---

INTEMPERANCE.—Dr. Druitt, of London, finished an address before the Health officers recently with the following conclusion:—1. That the secret drinkers, for the most part, may be restored by kind medical treatment. 2. That public drinking can only be put down by improved public opinion, education, and circumstances. 3. That every possible restriction be put upon the sale of spirits, especially on Sundays, and that power be given to the ratepayers to veto the establishing or licensing of public houses. 4. That habitual drunkards be encouraged to become teetotallers. 5. That the teetotal system operates beneficially, not by the pledge, which is often broken, but by the system of lectures and other means of moral and theological excitement. 6. That it were wise policy to provide rational amusement and wholesome refreshment at cost price for the masses. 7. That open drunkards be punished. 8. That drunkenness, together with the lesser forms of insanity, extravagance, gambling, betting, violence of temper, and other ruinous indulgences, be subject to a Court of Chancery (?) at the instance of the persons on whom the care and maintenance of such drunkard, gambler, etc., would fall, in the event of ruin. 9. That open drunkards be punished, and houses in which drunkenness is permitted be shut up. 10. That the common education of all classes is defective in moral teaching, and in training in the practice of abstinence.—*Med. and Surg. Reporter.*

---

AGGREGATION IN THE DUBLIN LYING-IN HOSPITAL.—Dr. Matthews Duncan, (*Med. Press and Circular,*) investigated the history of the Dublin Lying-in Hospital, with a view to trace the effects of crowding the wards. The hospital has been in operation 100 years, and has received nearly 200,000 patients. When it was emptiest, and contained about 1000 beds, the mortality was 14 per 1000. When the patients numbered 2300, the deaths were 12 per 1000; when 2800, 14 per 1000; when 3300, 13 per 1000. He holds that these figures prove that aggregation in that hospital did not increase the mortality.—*Pacific Medical and Surgical Journal.*

THE NEW YORK ACADEMY OF MEDICINE.—The twenty-second anniversary of the Academy of Medicine, New York, was held in the College of Physicians, on the evening of October 11th. The address was delivered by the President, Gouverneur M. Smith, M.D. In the course of his remarks he said:—

"Of the thirteen Fellows who have presided over it since its inception in 1846, nine have been gathered to their fathers; and it is a noteworthy fact, strongly disproving the frequent assertion that medical science tends to materialism and infidelity, that they all died rejoicing in Christian faith and hope. Since the last anniversary, death has also removed from the Academy, O'Reilly, Enos, Stevens, Guilford, 'men who would have adorned any calling, and of whom the profession has reason to be proud.'"—*Med. and Surg. Reporter.*

